# Cerebrospinal Fluid Analysis in Multiple Sclerosis Diagnosis: An Update

**DOI:** 10.3390/medicina55060245

**Published:** 2019-06-04

**Authors:** Bruna Lo Sasso, Luisa Agnello, Giulia Bivona, Chiara Bellia, Marcello Ciaccio

**Affiliations:** 1 Institute of Clinical Biochemistry, Clinical Molecular Medicine and Laboratory Medicine, Department of Biomedicine, Neuroscience and Advanced Diagnostics, University of Palermo, 90100 Palermo, Italy; bruna.losasso@unipa.it (B.L.S.); luisa.agnello@unipa.it (L.A.); giulia.bivona@unipa.it (G.B.); chiara.bellia@unipa.it (C.B.); 2 Department Laboratory Medicine, University-Hospital, 90100 Palermo, Italy

**Keywords:** multiple sclerosis, oligoclonal bands, demyelinating diseases, biomarkers, immunoglobulin light chains, cerebrospinal fluid

## Abstract

Multiple sclerosis (MS) is an immune-mediated demyelinating disease of the central nervous system (CNS) with brain neurodegeneration. MS patients present heterogeneous clinical manifestations in which both genetic and environmental factors are involved. The diagnosis is very complex due to the high heterogeneity of the pathophysiology of the disease. The diagnostic criteria have been modified several times over the years. Basically, they include clinical symptoms, presence of typical lesions detected by magnetic resonance imaging (MRI), and laboratory findings. The analysis of cerebrospinal fluid (CSF) allows an evaluation of inflammatory processes circumscribed to the CNS and reflects changes in the immunological pattern due to the progression of the pathology, being fundamental in the diagnosis and monitoring of MS. The detection of the oligoclonal bands (OCBs) in both CSF and serum is recognized as the “gold standard” for laboratory diagnosis of MS, though presents analytical limitations. Indeed, current protocols for OCBs assay are time-consuming and require an operator-dependent interpretation. In recent years, the quantification of free light chain (FLC) in CSF has emerged to assist clinicians in the diagnosis of MS. This article reviews the current knowledge on CSF biomarkers used in the diagnosis of MS, in particular on the validated assays and on the alternative biomarkers of intrathecal synthesis.

## 1. Introduction

Multiple sclerosis (MS) is a chronic inflammatory neurodegenerative disease of the central nervous system (CNS) that affects young adults. MS, as well as most immune-mediated diseases, is characterized by a sexual dimorphism influencing both incidence and severity of the disease. While women show a higher incidence of disease (ratio of 2:1–3:1), male sex is associated with a more rapidly progressive clinical course [1,2]. The symptoms depend on the localization of inflammatory foci that can affect all regions of CNS and include altered memory, view and attention, and difficulty in daily activities. Both genetic and environmental factors have been implicated in the pathogenesis of the disease, whose common mechanism seems to be an inflammatory process limited to the CNS with destruction of myelin and oligodendrocytes [2].

MS affects more than 2.5 million people in the world, with an incidence that increases with latitudes, being minimal at the equator and increasing at northern or southern latitudes, identifying Northern Europe as a high-risk area (prevalence 140–250 per 100,000) [1,3,4,5,6,7]. This unusual distribution suggests a connection between exposure to sun rays and onset of pathology with a focus on vitamin D status and its latitude-related synthesis. Several studies have showed an association among hypovitaminosis D, genetic polymorphisms in the vitamin D metabolism, and an increased incidence of MS [8,9,10,11,12,13].

**Figure 1:**
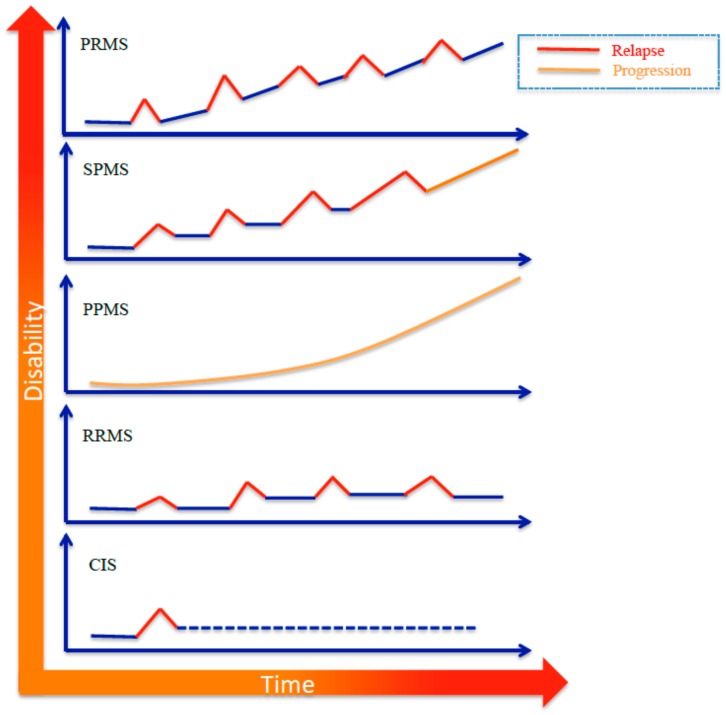
Symptom patterns that define the subtypes of multiple sclerosis (MS). CIS, clinically isolated syndrome; RRMS, relapsing/remitting multiple sclerosis; PPMS, primary progressive multiple sclerosis; SPMS, secondary progressive multiple sclerosis; PRMS, progressive-relapsing multiple sclerosis.

Clinical manifestations of MS are characterized by a high inter-individual variability with severity and duration of symptoms depending on the extension of the neurological damage. Indeed, periods of remission from the disease can be followed by relapses, defined as acute or subacute clinical episodes with neurological abnormality of at least 24 h. The Expanded Disability Status Scale (EDSS) score quantifies disability in MS, and it is commonly used to monitor disease progression. MS is categorized into four distinct subtypes, primarily based on its clinical course, which are characterized by increasing severity: (a) Relapsing remitting MS (RRMS), the most common form, affecting 85% of all MS patients, which involves relapses followed by remission; (b) secondary progressive MS (SPMS), which develops over time following diagnosis of RRMS; (c) primary progressive MS (PPMS), which is characterized by a gradual continuous neurologic deterioration; and (d) progressive relapsing MS (PRMS), which is similar to PPMS but with overlapping relapses (Figure 1) [2].

The first clinical presentation of MS is the clinically isolated syndrome (CIS). CIS refers to a neurologic episode that lasts at least 24 h, caused by inflammation or demyelination. If CIS is accompanied by MRI findings that show a past episode had occurred, an MS diagnosis can be made. However, not all patients convert from a CIS form to clinically definite MS. Validated biomarkers are therefore essential to predict the relapse and to identify the progression to different MS subtypes.

## 2. Etiopathogenesis

Etiology and pathogenesis of MS are still unclear. However, the immune system certainly plays an important role. It is well known that the intrathecal synthesis of immunoglobulins (Ig), expression of an altered immune response by T cells and plasma cells, causes CNS inflammation, loss of the myelin, and oligodendroglia cell death. B cells are protagonists of the adaptive humoral immune system and are responsible of the production of antigen-specific immunoglobulin. Immunoglobulins are assembled by the combination of five types of heavy chains (μ, δ, γ, α, and ε), and of two types of light chains (κ and λ). The five main classes of immunoglobulins are IgG, IgA, IgD, IgE, and IgM. IgG are the most abundant immunoglobulins in cerebrospinal fluid (CSF). Normally, the light chains are produced in excess of heavy chains [14,15]. Therefore, after the assembly of the complete immunoglobulin, the free light chains (FLCs) are released in the blood. The serum FLC concentration is low, as well as in urine (<15 mg/L), with the exception of lymphoproliferative disease and malignant disease in which Bence Jones proteinuria is usually present [16,17,18]. Several studies have documented the presence of B cells in white matter lesions of MS subjects, an increased CSF concentration of B cell-related cytokines (e.g., CXCL13 and BAFF), and myelin-specific antibodies. Moreover, such patients showed a good therapeutic response to the B cell depletion [19,20,21]. Myhr, K.M. et al. have recently highlighted that the depletion of B cells suppressed the inflammatory processes in RR-MS and slowed progression into more aggressive forms (PPMS) [22].

## 3. Diagnostic Criteria

The diagnosis of MS is very complex due to the heterogeneity of the disease. Diagnostic criteria rely mainly on the evaluation of clinical, imaging, and laboratory findings described in the “McDonald Criteria”. These criteria have been verified in clinical practice, validated, and progressively updated over the years (2005, 2010, and 2017) in consideration of the difficulties of an early identification of the disease that highlights the need of new technologies for an objective evaluation of patients with suspected MS [23,24,25].

Since the first definition of the McDonald criteria, the need to identify a “dissemination” of lesions in space and time has been emerged. The dissemination in space (DIS) is the development of lesions in distinct anatomical areas within the CNS. Dissemination in time (DIT) is represented by the development of new CNS lesions over time. The identification of DIS and DIT is important because the single lesion is not sufficient to diagnose MS, as well as a single episode of the disease over time. The update of McDonald criteria underlines the need to differentiate the first clinical episode (clinically isolated syndrome, CIS) from other neurological diseases, evaluating clinical signs, the objective finding of DIS and DIT by MRI, and laboratory findings (Table 1). In particular, the presence of CSF-specific IgG oligoclonal bands (OCBs) represents the gold standard for the diagnosis of MS [25].

## 4. CSF Diagnostics

The CSF analysis has a great potential as a source of emerging biomarkers for neurodegenerative diseases because it offers the opportunity to directly evaluate specific CNS inflammatory processes and to identify changes in the immunological pattern due to the progression of the disease [26,27]. The McDonald criteria emphasize that the CSF examination is a “valuable diagnostic test” especially when clinical evaluation and imaging (magnetic resonance imaging, MRI) don’t provide sufficient evidence to support the diagnosis of MS, or in presence of PP-MS, or in populations in which MS has a low prevalence (e.g., elderly, children, or non-Caucasian ethnic groups) [25].

**Figure 2:**
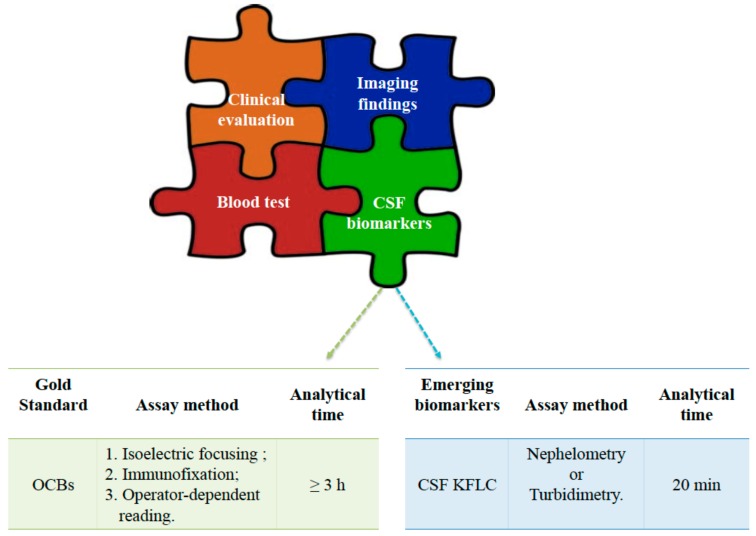
Diagnosis of multiple sclerosis with analytical differences between detection of CSF kappa free light chains (kFLC) *versus* oligoclonal bands (OCBs) assay.

Intrathecal positivity of immunoglobulins can be observed in some diseases of the CNS and can be attributable to several mechanisms, such as an altered permeability of the blood–brain barrier, an intrathecal synthesis of immunoglobulins, or a combination of both [28,29]. From a pathophysiological point of view, it is of great interest to distinguish the intrathecal synthesis of Ig from those “poured” back into the CSF through the blood–brain barrier (BBB). During inflammatory processes, B cells can migrate into the CNS, producing Ig. The newly synthesized Ig contribute to the CSF pool of Ig, including the ones derived by BBB diffusion [30,31]. In this context, the examination of CSF includes the quantitative measurements of Ig and albumin and a qualitative analysis of CSF Ig in comparison with serum Ig. The latter is based on the detection of oligoclonal bands by isoelectric focusing (IEF) followed by IgG specific immunoblotting (Figure 2). The detection of a specific IgG oligoclonal profile in CSF, not detected in serum, remains the major biochemical diagnostic marker for MS because it specifically indicates intrathecal synthesis of IgG [32,33]. However, this approach has several limitations, such as the operator-dependent interpretation of results; the need of time-consuming protocols, with an average time for analytical processing of over 3 h; and high costs. Moreover, there is no consensus on the definition of cut-offs for positivity. It should also be noticed that OCBs can be observed in patients affected by other neurological diseases, and they can be detected in about 8% of individuals from the general population [33,34,35,36,37].

In this context, the quantification of the kappa free light chain (kFLC) and the λ free light chain (λFLC) in CSF seems to be a promising test [37,38,39] (Figure 2). Most authors have focused their interest on kFLC chains compared to λFLC. This choice is supported by the observation of a higher increase of kFLC in subjects with MS compared to λFLC in CSF, suggesting more suitability for clinical purposes [35,39].

Recently, the clinical use of the K Index [(CSF_FLC_/Serum_FLC_)/(CSF_Albumin_/Serum_Albumin_)] has been proposed instead of the CSF kappa chain determination alone because the K Index takes into account the BBB integrity and it showed a higher diagnostic accuracy with a lower rate of false positives results [37]. The recent interest in FLC has been mainly supported by the availability of feasible and automated assays. The most common assays used for the measurement of FLC are turbidimetry or nephelometric assays (Figure 2) [14,29,40,41]. In CNS infectious diseases, an increase of λFLC levels was observed, and in some cases it was greater than the one observed for kFLC. Thus, an increase in λFLC could be suggestive of infection in those cases in which the diagnosis of MS is not well defined [15]. Mass spectroscopy studies have shown that the kappa chains may be detected in CSF as monomers and dimers, while the λ chains are mainly in the form of dimers [42]. Ramsden et al. argued that the presence of multiple states of aggregation of the analyte could affect quantification when nephelometric assays, which are strongly influenced by the rate of antibody-antigen aggregation, are used. At present, this issue requires more investigations due to conflicting data from literature [37,38,41,43]. Recently, Nazarov, V. et al. showed an association between kFLC and the degree of irreversible disability in MS patients. In particular, the authors showed that MS patients with high levels of kFLC reached disability faster than patients who had low kFLC levels, suggesting that it can be a good prognostic marker in MS [44,45]. Goffette et al. studied 33 patients with clinically suspected MS and no detectable OCB and reported that 54% of them were positive for kFLC. The Authors concluded that the detection of CSF kFLC could replace the CSF-specific IgG OCB [46]. Valencia-Vera, E. et al. have recently evaluated the diagnostic accuracy of K Index in 123 consecutive subjects undergoing the CSF OCB test and calculated an algorithm including both K Index and OCB interpretation. The authors evaluated the kFLC assay as a screening tool for the selection of patients that require OCB test for diagnosis confirmation. Nevertheless, the authors failed to demonstrate a higher diagnostic sensitivity and specificity of kFLC in comparison to OCB test [32]. It should be emphasized that the published studies on this topic examined small populations and used different analytical methods, metrics, and cut-offs (Table 2) [20,32,46,47,48,49,50].

Therefore, the clinical use of kFLC requires further investigations in order to obtain a definitive validation.

The actual McDonald criteria recommend the analysis of oligoclonal IgG bands for the evaluation of patients with suspected MS but didn’t include the quantification of kFLC as a possible diagnostic biomarker [25]. Moreover, the updated criteria emphasize the lack of a single laboratory test with adequate diagnostic performance for the diagnosis of MS. Nevertheless, at present, differential diagnosis between suspected MS and other CNS demyelinating pathologies can be made using serological evaluation of the IgG autoantibodies anti-aquaporin-4 (AQP4) and the IgG autoantibodies directed against the oligodendrocytic myelin glycoprotein (MOG-IgG). Both these tests are useful when clinical presentation, imaging, or laboratory findings are atypical in MS suspected patients. In particular, the AQP4 autoantibody is a specific biomarker for neuromyelitis optica spectrum disorders (NMOSD) [51]. MOG-IgG antibody is a biomarker of MOG-associated encephalomyelitis (MOG-EM), a relatively rare demyelinating disease characterized by different clinical manifestations, including recurrent and bilateral optic neuritis, transverse myelitis, brain stem encephalitis, and acute disseminated encephalomyelitis (ADEM) [25,52,53].

## 5. Conclusions

The CSF FLCs analysis reflects the intrathecal synthesis of immunoglobulins. At present, their clinical utilization is limited by analytical factors, the absence of reference values and clinically-validated cut-offs [53]. On the other hand, the evaluation of OCB, considered the gold standard for the demonstration of Ig intrathecal synthesis by international guidelines, is a time-consuming and laborious assay affected by an operator-dependent interpretation. A misleading interpretation of CSF findings can occur, especially when ambiguous oligoclonal band patterns are detected. In these cases, the MS diagnosis should be considered with caution and the introduction of a more accurate biomarker is advocated [54]. The integration among physical examination, imaging, and an appropriately validated intrathecal FLC index, together with CSF OCBs represents a promising strategy to guide clinicians towards an early diagnosis of MS and adequate treatments in order to improve the quality of life of MS patients and limit their disability.

## Figures and Tables

**Table 1:** 2017 McDonald criteria (2017) for the diagnosis of MS.

**Clinical Presentation**	**Additional Data Needed for MS Diagnosis**
In patient with a typical attack/CIS at onset	
≥2 clinical attacks and evidence of ≥2 lesions	None *
≥2 clinical attacks and evidence of 1 lesion with history of previous attack involving lesions in different location	None *
≥2 clinical attacks and evidence of 1 lesion	Evidence for DIS established by an additional clinical attack implicating different CNS site OR by MRI
1 clinical attack and evidence of ≥2 lesions	Evidence for DIT established by an additional clinical attack implicating different CNS site or by MRI OR positive CSF-specific (i.e., not in serum) oligoclonal bands **
1 clinical attack and evidence of 1 lesion	Evidence for DIS established by an additional clinical attack implicating different CNS site or by MRI ORevidence for DIT established by an additional clinical attack implicating different CNS site OR by MRI OR positive CSF-specific (i.e., not in serum) oligoclonal bands **
**Clinical presentation**	**Additional data needed for MS Diagnosis**
In patient with a progression of disability from onset (PPMS)	
Progression from onset	1 year of disability progression (retrospective or prospective) AND two of these criteria: ≥ 1 symptomatic and asymptomatic T2-hyperintense lesions (periventricular, cortical or juxtacortical, or infratentorial); ≥ 2 T2-hyperintense lesion in spinal cord; Positive CSF-specific (i.e., not in serum) oligoclonal bands **

* No additional data are required to demonstrate DIS and DIT. ** Positive CSF-specific oligoclonal bands (OCBs) are defined by the presence of at least two CSF-specific bands. MS, multiple sclerosis; CIS, clinically isolated syndrome; DIS, dissemination in space; DIT, dissemination in time; CNS, central nervous system; MRI, magnetic resonance imaging; CSF, cerebrospinal fluid; PPMS, primary progressive multiple sclerosis.

**Table 2:** Clinical sensitivity and specificity of metrics in multiple sclerosis. MS, multiple sclerosis; CIS, clinically isolated syndrome; OCB, oligoclonal bands; CSF kFLC, Cerebrospinal fluid K free light chain; CSF λFLC, Cerebrospinal fluid λ free light chain.

Authors [Ref.]	Number of Subjects	Assay Method	Metrics	Cut-Off	Sensitivity, %	Specificity, %
Menendez-Valladares, P. et al. [50]	334 patients	Nephelometry	kFLC Index	10.62	91%	89%
Saez, M.S. et al. [47]	77 patients	Turbidimetry	OCB	Positive	93%	90.4%
			CSF kFLC	7.1 mg/L	95%	97%
			CSF λFLC	0.7 mg/L	71%	81%
			kFLC + λFLC	/	95%	
Puthenparampil, M. et al. [20]	70 patients	Nephelometry	IgG Index	/	/	/
			CSF and serum kFLC	/	/	/
	37 controls		CSF and serum λFLC	/	/	/
			kFLC Index	4.25	93.8%	100.0%
			λFLC Index	/	/	/
Christiansen, M. et al. [48]	230 patients	Turbidimetry	OCB	Positive	82.3% (MS) 56.8% (CIS)	93.8%
			IgG Index	0.64	72.9% (MS) 51.3% (CIS)	95.9%
			CSF kFLC	0.42 mg/L	93.8% (MS) 70.3% (CIS)	85.6%
			CSF λFLC	0.14 mg/L	93.8% (MS) 86.5% (CIS)	35.1%
			kFLC Index	5.9	92.7% (MS) 70.3% (CIS)	86.6%
			λFLC Index	2.8	93.8% (MS) 81,1% (CIS)	46.4%
Gurtner, K.M. et al. [49]	325 residual paired CSF and serum samples	Nephelometry	OCB	Positive (≥4 bands)	86.6%	89.6%
			CSF kFLC	0.0611 mg/dL	92.5%	86.1%
			CSF λFLC	0.0244 mg/dL	75.8%	84.4%
			kFLC Index	≥8.87	88.1%	88.7%
Valencia-Vera, E. et al. [32]	123 unselected consecutive patients with CSF OCB request	Nephelometry	OCB	Positive	89.2%	81.2%
				≥0.92	97.3%	45.88%
			kFLC index	≥2.91	83.8%	85.8%
				≥8.33	70.2%	95.6%
Goffette et al. [46]	33 patients with clinical suspicion of MS	Immunoaffinity-mediated capillary blot	Free K Bands	Presence of free kappa bands	/	/
